# How Should Anesthesiologists Face Coronavirus Disease 2019?

**DOI:** 10.3389/fcvm.2022.890967

**Published:** 2022-05-27

**Authors:** Long Feng, Shihui Fu, Pei Zhang, Yao Yao, Zeguo Feng, Yali Zhao, Leiming Luo

**Affiliations:** ^1^Department of Anesthesia, Hainan Hospital of Chinese People's Liberation Army General Hospital, Sanya, China; ^2^Cardiology Department, Hainan Hospital of Chinese People's Liberation Army General Hospital, Sanya, China; ^3^Department of Geriatric Cardiology, Chinese People's Liberation Army General Hospital, Beijing, China; ^4^School of Life Science, Beijing Institute of Technology, Beijing, China; ^5^Center for the Study of Aging and Human Development and Geriatrics Division, Medical School of Duke University, Durham, NC, United States; ^6^Center for Healthy Aging and Development Studies, National School of Development, Peking University, Beijing, China; ^7^Department of Pain, First Medical Center of Chinese People's Liberation Army General Hospital, Beijing, China; ^8^Central Laboratory, Hainan Hospital of Chinese People's Liberation Army General Hospital, Sanya, China

**Keywords:** anesthesia, anesthesiologist, coronavirus disease 2019, intubation, perioperative period, personal protective equipment

## Abstract

Coronavirus disease 2019 (COVID-19) is a highly contagious disease. Most infected patients manifest mild flu-like symptoms, but in some cases, the patients rapidly develop severe lung infections and pneumonia. It is estimated that about 15–20% of patients with COVID-19 develop hypoxemia and require some form of oxygen therapy and ventilation support. Further, exacerbation of the disease usually requires an emergency tracheal intubation, where the patients are more prone to coughing and aerosol diffusion, placing the anesthesiologist at an extremely high risk of infection. In this review, after a brief introduction to the epidemiology and pathogenesis of the COVID-19, we describe various recommendations that the anesthesiologists should employ to avoid the chances of infection during the management of severely ill patients. We describe key steps such as not removing the patient's mask prematurely and using sedatives, analgesics, and muscle relaxants for rapid and orderly intubation. The use of spinal cord and regional nerve block anesthesia should also be promoted to avoid general anesthesia. Since the patients with COVID-19 may also have disorders related to other parts of the body (other than lungs), short-acting drugs are recommended to actively maintain the perfusion pressure of the peripheral and important organs without metabolism of the drugs by the liver and kidney. Multimodal analgesia is advocated, and non-steroidal anti-inflammatory analgesic drugs can be used appropriately. In this review, we also discuss key studies and experiences of anesthesiologists from China, highlights research findings, and inform on the proper management of patients with perspective on anesthesiologists.

## Introduction

Coronavirus disease 2019 (COVID-19) is a highly infectious disease that is caused by a new coronavirus. Most infected patients manifest mild symptoms such as fever, fatigue, and cough; however, in some cases, the disease can be severe, where the patients rapidly develop acute respiratory distress syndrome (ARDS), septic shock, metabolic acidosis, and coagulopathy (1).

In a retrospective study, it was found that approximately 15–20% of patients with COVID-19 developed hypoxemia and required some form of oxygen therapy and ventilation support (2). Emergency endotracheal intubation, due to dyspnea and decreased oxygen saturation, may also be required during the disease exacerbation. Since patients are more likely to cough and diffuse aerosols during the tracheal intubation, anesthesiologists are at a very high risk of infection (3). As airway management experts, anesthesiologists are among the first-line participants involved in the management of critically ill patients with COVID-19. Hence, it is important for anesthesiologists to prepare for protection in different medical procedures, and identify safer and more appropriate anesthesia methods. In this review, we summarize the epidemiological and pathological features of COVID-19 and current practices of self-protection, tracheal intubation, and choice of anesthesia methods employed by anesthesiologists from China during the management of COVID-19.

## Epidemiological and Pathological Features

Coronaviruses constitute a large group of viruses in the order Nidovirales (family Coronaviridae and subfamily Orthoviridae); they are positive single-stranded enveloped viruses, and the particles are round or oval, usually polymorphic, with a diameter of 50–200 nm (4). The spike (S) structural proteins are located on the surface of the virus and form a rod-like structure. The S proteins are the main antigenic proteins of the virus, and are required for host entry (5). Fu et al. (6) reported that 2019 novel coronavirus (2019-nCoV) S protein binds to the human angiotensin-converting enzyme 2 (ACE2) molecules, and hence, the virus causes a major public health risk. Coronaviruses spread widely in humans and other mammals and birds, and lead to acute respiratory diseases (7). Respiratory droplets are the main route of COVID-19 transmission; human to human transmissions mainly occur in close contacts such as family members, relatives, and friends who are either patients or carriers. Emerging evidence also suggests that 2019-nCoV can also transmit through the fecal-oral route. Further, it has been demonstrated that COVID-19 imposes a heightened risk for subsection of population (8, 9), for instance, elderly patients with comorbid diseases such as asthma, heart diseases, diabetes, and immunodeficiency diseases. Since smoking imposes a negative and harmful effect on the immune system of the body, smokers are generally considered to be more susceptible to such infectious diseases. Furthermore, pregnant women observe different physiological modifications in the immune and cardiopulmonary systems that may expose them to the adverse consequences of viral respiratory infections (10).

Coronaviruses are heat-sensitive and can be killed by treatment at 56°C for 30 min. Chemical treatments with ether, 75% ethanol, chlorine disinfectant, peracetic acid, or chloroform can also effectively inactivate the virus; however, chlorhexidine treatment is sufficient to inactivate the virus (11). Chen et al. (11) and Huang et al. (12) reported that in confirmed COVID-19 cases, common symptoms were fever, cough, pneumonia, dyspnea, lymphopenia, and muscle pain. In severe COVID-19 cases, patients often show dyspnea with a respiratory rate of 30 breaths per min, 93% finger oxygen saturation at rest, and PaO_2_/FiO_2_ ratio of 300 mmHg Column (1 mmHg = 0.133 kPa; PaO_2_, arterial partial pressure of oxygen; FiO_2_, fractional concentration of inhaled oxygen) (1). Furthermore, to manage disease exacerbations, ventilation support system is required that includes the high-flow oxygen therapy, non-invasive ventilation, invasive mechanical ventilation, and extracorporeal membrane oxygenation. It is also reported that intubation should not be delayed in patients with viral pneumonia and acute respiratory failure (13). Psychological factors in the epidemic environment also play crucial role; people often suffer from anxiety, restless sleep, sadness, irritability, anger, and violence (14). Inflammatory cytokines are a major factor in the pathogenesis of stress, anxiety, depression, and other mental diseases (15). In the state of anxiety, the proliferation of T cells and the body immunity is reduced (16). Therefore, in this critical situation (COVID-19 pandemic), psychologists may play a very important role in caring about people's mental health and providing necessary measures to reduce people's stress (1).

## Personal Protection for Anesthesiologists

The COVID-19 infection is mainly transmitted through the respiratory secretions and aerosols, and hence, while performing tracheal intubation of suspected or confirmed patients, the personal protective equipment (PPE) for the healthcare workers in the anesthesia preoperative evaluation clinics should include white medical gowns, medical gloves, eye protection shields, disposable surgical caps, and surgical masks or test-fit N95 masks or respirators (17). Further, double glove technique on both hands should be employed (18). For anesthesiologists or personnel engaged in aerosol-generating medical treatments (tracheal intubation, non-invasive ventilation, tracheostomy, cardiopulmonary resuscitation, and bronchoscopy before manual ventilation intubation), standard stringent PPE such as electric air-purifying respirators may also be implemented (19, 20). In addition, training and awareness on evolving situation and facts of COVID-19 should be provided for better and safe management of severe acute respiratory diseases.

A previous study has reported the successful management of COVID-19 cases without cross-infection after the procedures (21). The specific prevention and control measures are as follows: (1) dedicated negative pressure patient transfer vehicles and operating rooms were used to prevent the spread of virus, and all the instruments and equipment such as anesthetic machine, cart, and monitor in the operating rooms were sterilized before and after the procedures, (2) established biosafety procedures were followed during the entry and exit of patients and paramedics, and (3) patients used mask while coughing during the tracheal intubation and suction to avoid contamination of the respirator. In addition, a gap of at least an hour between each operation was ensured. All unused items on medication trays and airway carts were assumed contaminated and discarded, and all doctors had a bath before returning to the normal work. It is also recommended that as a supplementary precaution, after confirming the case of COVID-19, the hydrogen peroxide vaporizer may be used to clean the operating rooms (22). Recent literature also indicates that the surgery-specific guidelines for COVID-19 emergencies may also be effective in preventing surgery for patients with suspected or confirmed COVID-19 (23).

Vaccination against 2019-nCoV is also an important protective step for anesthesiologists (24) because evidences suggest that vaccination provided improved adequate immunity against 2019-nCoV. Further, while being infected, fully vaccinated health care workers may often do not show serious symptoms due to presence of neutralizing antibodies (25). However, vaccination may not possible in all healthcare workers due to personal or religious objections, and hence, unvaccinated workers may be excluded from paramedical work to manage patients with confirmed COVID-19.

## Preanesthesia Assessment and Preparation

The incubation period of 2019-nCoV usually ranges from 1 to 14 days (1). The pre-anesthesia assessment of suspected or confirmed COVID-19 cases must be conducted mainly through the epidemiological investigations of the history of exposure to affected area, preoperative nucleic acid testing, body temperature, chest computed tomography (CT), and routine blood tests. Airway assessment is the most important part of the preoperative anesthesia evaluation, and tracheal intubation tools and medicines should be fully prepared before the procedures. Pre-anesthesia visits are best performed by analyzing the electronic medical records and telephonic pre-operative visits, while protection should be employed for patients who require face-to-face visits. Furthermore, rescue vehicles, blood gas analyzers, and other equipment can be placed in the operating rooms in advance to avoid cross-infection in the entire operation department.To evaluate the anesthesia risk of such patients before the procedures, special attention should be paid to the arterial blood gas analysis and pulmonary function assessment (23).

## Tracheal Intubation for Emergency

Open suctioning of the respiratory tract, manual ventilation before intubation, nebulizer treatment, and chest compressions were identified as the main risk procedures during the COVID-19 outbreak (26). It is necessary to avoid coughing during the economic tracheal intubation for such patients. For the better management, anesthesiologist who has experience with difficult airways during the tracheal intubation should be recruited. It has also been postulated that three levels of protection must be implemented before the intubation procedures (17). In a well-known story of a team that was recognized as the “coronavirus intubation team racing against death,” eighteen anesthesiologists and two anesthesia nurses from five different hospitals formed a garrison team and performed nearly 50 intubations for critically ill patients with COVID-19 over a period of 8 days (27). Such teams can be prepared in advance to improve the success rate of intubation and prevent cross-infection among the other medical staff. For departments that often require tracheal intubation and rescue (such as emergency rooms and intensive care units), a set of special intubation tools can be equipped to avoid cross-infection with anesthesia surgery departments.

In general, the recommended procedure guidelines state that the patient's mask should not be removed as much as possible during the tracheal intubation, and sedatives, analgesics, and muscle relaxants should be administered to induce intubation. Rocuronium, propofol, and sufentanil are commonly used drugs before the intubation procedures; sufentanil (1 mL; 10 μg) and rocuronium (5 mL; 50 mg) should be provided together to avoid multiple administrations. The dosage of propofol (generally 20–50 mg) is administered after comprehensive evaluation based on the patient's age, weight, and circulation condition. Intratracheal intubation is usually performed 1–2 min after the drug administration. Etomidate is also an option for fragile and elderly patients. Furthermore, to avoid close contacts with the patient's oropharynx, use of visual laryngoscope for intubation is recommended. It is best to avoid the use of Airtraq video laryngoscopes as proximity to the patient's nose and mouth greatly increases the risk of infection.

## Precaution for Anesthesia Management

It is recommended that intubation procedures should be conducted in the operating rooms under a negative pressure. Expert consensus of the Chinese Association of Anesthesiologists states that when general anesthesia is necessary, a rapid induction of anesthesia and adequate relaxation of muscles are recommended to prevent coughing. The induction sequence is muscle relaxants, intravenous general anesthesia, and avoidance of opioid cough suppressant. Further, it is recommended to avoid the mask ventilation before the patients fall asleep (28). General anesthesia did not have any adverse effects on the newborn and the mother recovered from COVID-19 (21). For such patients, extubation should be performed under deep anesthesia to avoid coughing. These patients often show diffuse alveolar injury accompanied by hyaline membrane formation, infiltration of monocytes and macrophages in the alveolar cavity, and diffused thickening of the alveolar wall in the early and late lung pathology (29, 30). The infected alveolar cells cause the expansion of alveolar capillaries, the increased permeability of microvessels and alveolar walls, the reduction of pulmonary surfactant and the collapse of alveoli, the loss of the gas exchange function caused by the flight, which leads to the formation of ARDS (31). Therefore, it is important to perform effective oxygenation and lung protective ventilation strategies for these patients during the procedures. A small tidal volume added positive end-expiratory pressure (PEEP) protective ventilation strategy should be used during the anesthesia to reduce ventilator-related lung injury. Tidal volume is generally set to 4–8 mL/kg body weight, while the suction platform pressure is <30 cm H_2_O, and PEEP level is <8 cm H_2_O (32).

In addition to lung injury, COVID-19 also lead to damage in other organs such as the liver, kidney, heart, brain, and systemic immune system. Because kidney cells express high levels of ACE2 receptors, it is an important target of 2019-nCoV. In a study, twenty seven percent of the total 85 hospitalized patients also had renal failure. Among them, patients with acute kidney injury showed more than five times mortality than that of the patients without such injury (31). Furthermore, bile duct cells express high levels of ACE2 receptors, which is more than 20 times than that of the stem cells. Compared with patients with normal liver function on admission, those with mixed liver dysfunction (4.44 times more) had a high risk of developing severe disease (32). The research team also pointed out that patients with abnormal liver function are more likely to develop a more serious disease state, and the use of drugs during the hospitalization may cause liver damage, and should be closely monitored (33). Therefore, during the anesthesia, liver and kidney non-metabolizable drugs should be used as much as possible (such as muscle relaxants cisatracurium). In addition, the perfusion pressure of important organs should be maintained as high as possible during the operation. Further, a close attention should be made to the amount of urine during the operation, especially when the bleeding is heavy. Most patients with severe COVID-19 also show risk of acute myocardial injury. In addition, the underlying cardiovascular disease may be a contributing factor to the condition of COVID-19 (34). It is suggested to actively treat the primary disease and carry out myocardial protection treatment for acute myocardial injury associated with COVID-19. For the anesthesia of these patients, maintaining the stability of the circulation as much as possible is crucial. Further, depending on the requirement, positive inotropic and myocardial protective drugs can be administered.

It is also believed that 5–10% of patients with COVID-19 had neurological symptoms, which may be related to the presence of ACE2 receptors in the cortex and brain stem (31). Further, patients with complicated conditions of meningitis and encephalitis are also observed, indicating that 2019-nCoV can invade the central nervous system and cause meningitis or encephalitis. For such patients, drugs (such as propofol or dexmedetomidine) with certain anti-inflammatory brain protection should be used during the procedures. Besides, most patients with severe COVID-19 exhibit substantially elevated serum levels of pro-inflammatory cytokines, characterized as the “cytokine storm.” C-reactive protein and D-dimer are also found to be abnormally high. High levels of pro-inflammatory cytokines may lead to shock and tissue damage in the heart, liver and kidney, as well as respiratory failure or multiple organ failure (35). For the anesthesia of these patients, adequate analgesics should be given to prevent excessive pain stress reactions from aggravating systemic inflammatory reactions. It is best to implement a multi-modal analgesic method during the perioperative period (cyclooxyganese-1 and cyclooxyganese-2 non-steroidal anti-inflammatory analgesic drugs) and hormones should be used appropriately (prednisolone, dexamethasone, etc.).

At present, Chinese domestic treatment experience and guidelines recommend the spinal canal anesthesia or peripheral nerve block as much as possible (18); however, for patients who must undergo general anesthesia, all protection strategies must be employed. The breathing loop of the ventilator preferably uses three artificial noses at the gas inlet and outlet and behind the patient's mask during the anesthesia. The use of artificial nose can prevent the anesthesia machine from being infected by the virus. It is generally recommended to use an artificial nose with a filtering function, and a simple filter or a composite artificial nose (heat and moisture exchange + filter), both of which have filter membranes.

Epidural and general anesthesia was found to be safe for neonatal pneumonitis in cesarean section (21). Nonetheless, the incidence of hypotension during the epidural anesthesia may be due to COVID-19 binding to ACE2 receptors. The key to COVID-19 infection is its S protein binding to the ACE2 receptors (36, 37). Li et al. (38) also pointed out that in clinical work, even if these patients do not show hypotension, many severe or critically ill patients often have typical shock symptoms such as cold limbs and weak pulse, and some of them also show metabolic acidosis, which suggests that microcirculation dysfunction may occur. Hence, maintaining a high perfusion pressure during the induction and maintenance of anesthesia, preventing the occurrence of hypotension, and making a good insulation measure (such as the use of insulation blankets or liquid warmers) are necessary. Patients with advanced coagulation function can develop abnormalities in the late course of COVID-19, especially significantly increased D-dimer levels (39). In a study, 38% of the total 184 in-patients showed abnormal coagulation function, and plaque shedding may cause pulmonary embolism or stroke (31). Thus, during the spinal anesthesia or nerve block, monitoring of the coagulation function of patients is crucial to prevent complications such as epidural hematoma. In addition, correct patient transfer, medical staff access procedures, and effective biosafety precautions are vital to protecting lives from COVID-19. In addition, patients with COVID-19 should not remain in the recovery rooms (post-anesthesia care unit). After a complete recovery in the operating room, the patient should be transferred to the negative pressure room of a ward or intensive care unit (17).

## Conclusions

Anesthesiologists are airway management specialists in various medical units and often deal with severe patients on the front line. Because 2019-nCoV is transmitted through close contacts, all medical personnel, including anesthesiologists, are required to use level 3 PPE to protect themselves from infection, particularly during the tracheal intubation procedures in severely ill patients. The patient's cough during the tracheal intubation or the spread of aerosols after opening the mouth exposes the anesthesiologist to a high risk of infection, and hence, anesthesiologists should also evaluate the patient's airway conditions before tracheal intubation, prepare as much as possible in advance, allow patients to fully inhale oxygen, and avoid removing patient's masks before intubation. Further, to avoid patients from coughing, a rapid laryngoscope intubation under sequence induction should be conducted. In general, for medical units that focus on treating patients with COVID-19, it is recommended to set up a special emergency tracheal intubation team that also includes anesthesiologists. To avoid chances to infection from patients, it is best to choose spinal cord and regional nerve block anesthesia as much as possible. Intraoperative management should pay much attention to lung protection and prevention of hypotension. It is optimal to remove the tracheal tube under deep anesthesia to avoid coughing and the spread of secretions. Since the patients with severe COVID-19 often suffer from liver and kidney injury, acute myocarditis, coagulopathy, systemic inflammation, and brain damage, short-acting drugs should be selected during the perioperative period to actively maintain the perfusion pressure of the peripheral and important organs without metabolism of the drugs by the liver and kidney. For patients who have difficulty maintaining the circulatory stability, positive inotropic and vasoactive drugs may be actively administered. Multimodal analgesia is advocated, and non-steroidal anti-inflammatory analgesic drugs and hormones can be used appropriately. Further, excessive intubation and painful stimulation should be avoided to not aggravate the systemic stress response.

## Author Contributions

All authors contributed to the design of the study, review of the literature, and drafting of the manuscript. All authors have read and approved the manuscript.

## Funding

This work was supported by grants from the National Natural Science Foundation of China (81900357, 81941021), Military Medical Science and Technology Youth Incubation Program (20QNPY110), Excellent Youth Incubation Program of Chinese People's Liberation Army General Hospital (2020-YQPY-007), Natural Science Foundation of Hainan Province (2021-253, 821MS112), Military Medicine Youth Program of Chinese People's Liberation Army General Hospital (QNF19069), National Key R&D Program of China (2018YFC2000400), National S&T Resource Sharing Service Platform Project of China (YCZYPT[2018]07), Specific Research Fund of The Innovation Platform for Academicians of Hainan Province (YSPTZX202216), and Medical Big Data R&D Project of Chinese People's Liberation Army General Hospital (MBD2018030). The sponsors had no role in the design, conduct, interpretation, review, approval, or control of this article.

## Conflict of Interest

The authors declare that the research was conducted in the absence of any commercial or financial relationships that could be construed as a potential conflict of interest.

## Publisher's Note

All claims expressed in this article are solely those of the authors and do not necessarily represent those of their affiliated organizations, or those of the publisher, the editors and the reviewers. Any product that may be evaluated in this article, or claim that may be made by its manufacturer, is not guaranteed or endorsed by the publisher.
